# Transcriptome analysis of tomato (*Solanum lycopersicum* L.) shoots reveals a crosstalk between auxin and strigolactone

**DOI:** 10.1371/journal.pone.0201124

**Published:** 2018-07-25

**Authors:** Yihua Zhan, Yinchao Qu, Longjing Zhu, Chenjia Shen, Xuping Feng, Chenliang Yu

**Affiliations:** 1 State Key Laboratory of Plant Physiology and Biochemistry, College of Life Sciences, Zhejiang University, Hangzhou, China; 2 Vegetable Research Institute, Zhejiang Academy of Agricultural Sciences, Hangzhou, China; 3 Wenzhou Vocational College of Science and Technology, Wenzhou, Zhejiang, China; 4 College of Life and Environmental Science, Hangzhou Normal University, Hangzhou, China; 5 College of Life Sciences, China Jiliang University, Hangzhou, People’s Republic of China; Hainan University, CHINA

## Abstract

Auxin and strigolactone (SL) are two important phytohormones involved in shoot branching and morphology. Tomato (*Solanum lycopersicum* L.), a member of the Solanaceae family, is one of the most popular food crops with high economic value in the world. To seek a better understanding of the responses to exogenous hormones, transcriptome analyses of the tomato shoots treated with exogenous auxin and SL, separately or together, were performed. A total of 2326, 260 and 1379 differential expressed genes (DEGs) were identified under the IAA, GR24 and IAA+GR24 treatments, respectively. Network analysis pointed out two enriched interaction clusters, including “ethylene biosynthesis” and “photosynthesis”. Several ethylene biosynthesis and metabolism-related genes were up-regulated under both IAA and IAA+GR24 treatments, suggesting their involvement in the regulation of ethylene biosynthesis. Besides, auxin-SLs-triggered the expression of several *CAB* genes may lead to systemic increases in the induction of photosynthesis. Several auxin-activated metabolic pathways could be reduced by the GR24 treatment, indicated that the crosstalk between auxin and SLs may be involved in the metabolic regulation of tomato. Further analysis showed that SLs affect the responses of tomato shoots to auxin by inducing the expression of a series of auxin downstream genes. On the other hand, auxin regulated the biosynthesis of SLs by affecting the genes in the “Carotenoid biosynthesis” pathway. Our data will give us an opportunity to reveal the crosstalk between auxin and SLs in the shoots of tomato.

## Introduction

Tomato (*Solanum lycopersicum* L.), one of the highest consumed horticultural crops, has been treated as a model plant for the studies on the *Solanaceae* family plants a decade ago [1–3]. To obtain different desirable features, such as the resistances to pathogen infections and the tolerances to stresses, tomato plants are typically grafted [4,5]. Grafting is an ancient plant propagation technique that has been widely applied in agricultural production [6]. Formation of graft union requires the re-programming of a series of biochemical and structural processes and is controlled by a complex hormonal network [7]. Application of exogenous hormones to cutting shoots is an key step for a successful grafting [8]. Thus, seeking a better understanding of the responses of shoots to exogenous hormonal treatments has been a hot region recently.

Several phytohormones have been reported to be involved in graft process [6,9]. In tomato, rootstock-mediated changes in hormone levels are associated with leaf senescence, leaf area and crop productivity [10]. Auxin is essential for vascular reconnection at the early stage of graft union formation. For example, the cell-cell communications between two cutting stems depend on local auxin accumulation [11]. The expression levels of many auxin-related genes are altered in graft interface zone [12]. Recently, the auxin signaling pathway, including the *Auxin Response Factor* (ARF) family [13], the *Auxin/Indole-3-Acetic Acid* (AUX/IAA) family [14], the *TOPLESS* family [15], the *Small Auxin Up-regulated RNA* (SAUR) family [16], and the *Polar Auxin Transport* (PAT) genes [17], has been well identified in tomato. For example, the auxin receptor tomato TRANSPORT INHIBITOR RESPONSE1 was identified by Hao’s group in 2011 [18]. SlTIR1, together with several down-regulation of transcription factors, such as SlIAA9 and SlARF7, constituted whole auxin signaling pathway in tomato [19,20]. The previous studies provided massive valuable information for us to discuss the responses of tomato stems to auxin application.

Besides, strigolactone (SL) is another important phytohormone involved in shoot branching and shoot morphology [21]. SLs were originally extracted from plant roots and recognized as beneficial metabolites for plant growth [22]. Several repressors of the SL signaling in rice and *Arabidopsis* have been identified, pointing out a consistent SL signaling pathway in plants [23]. In tomato, SLs were derived from carotenoids, which was affected by different environmental conditions [24]. Various SLs with diverse structures have been identified in tomato [25]. Several genes, such as *SlCCD7* and *SlCCD8*, were reported to be involved in the rhizosphere signaling and plant architecture by affecting the biosynthesis of SL in tomato [26].

Application of exogenous GR24, a synthetic SL, could affect the hormone profile in plants, indicating the interactions between SLs and other hormones [27]. For example, the interaction between endogenous SLs and abscisic acid (ABA) plays an essential role during the parasitic weed *Phelipanche ramosa* infection process [28]. ABA may affect the SL signaling by regulating the biosynthesis of SLs [29]. Gibberellin is another hormone involved in the SL biosynthesis by regulating the gibberellin (GA) receptor GA-insensitive dwarf1 (GID1) and F-box protein GID2 [30]. Increasing evidences showed that there was a close relationship between auxin and SLs in plants. In *Arabidopsis*, SLs function as the downstream pathway of auxin to control bud outgrowth [31]. On the one hand, auxin may act through SLs to regulate apical dominance [32]. On the other hand, SLs manipulate the auxin pathway by affecting auxin transport inducing TIR1 transcription to increase auxin perception [33]. Moreover, SlIAA27 plays a role in the establishment of mycorrhizal symbiosis, and its silencing results to the down-regulation of three strigolactone synthesis-related genes, *NSP1*, *D27* and *MAX1* [34].

In our study, investigation of the transcriptional responses of tomato shoots to exogenous auxin and SLs, separately or together, has been performed. Our data will give us an opportunity to reveal the crosstalk between auxin and SLs in the shoots of tomato.

## Materials & methods

### Plant materials, treatments and sampling

Seedlings of tomato (*Solanum lycopersicum* L. cv ‘microTom’) was grown in a greenhouse at a thermoperiod of 25°C/20°C and a photoperiod of 14/10 hours (day/night). The location of the greenhouse is at Zhejiang Academy of Agriculture Science, Hangzhou, Zhejiang, China. Fertilizer and water management are carried out according to the standard practices.

For hormone treatments, 12 four-week-old uniformly growing seedlings were selected. All the seedlings were grouped into four groups (three repeats in each group). For hormone treatments, 10 seedlings were treated as a group. For the auxin treatment, 100 μM of indole-3-acetic acid (IAA) solution was daubed to the shoots of tomato seedlings for 3 h. For the strigolactone treatments, 10 μM of GR24 solution was daubed to the shoots of tomato seedlings for 3 h. For the IAA+GR24 treatment, a mixture solution of 100 μM IAA and 10 μM GR24 was daubed to the shoots of tomato seedlings for 3 h. The shoots from the untreated seedlings were used as a control group. Then, all the shoots were collected and immediately put into liquid N_2_.

### RNA isolation and Illumina sequencing

Total RNAs from the tomato shoots were extracted using TRIzol reagent (Takara, Dalian, China) according to its protocol. The RNAs were treated with RNase-free DNase I (NEB, Beijing, China) at room temperature to remove the rest of DNA. The quality of RNAs was determined using an Agilent 2100 Bioanalyzer (Santa Clara, CA, USA). Three independent samples were prepared for each sample group.

For libraries construction, mRNAs were extracted using cellulose containing oligo-dT and fragmented into small fragments. First-strand complementary DNA (cDNA) was synthesized using several random hexamer-primers, and second-strand was produced using DNA polymerase I. The purified cDNA fragments were ligated with sequencing adapters and amplified by Polymerase Chain Reaction (PCR) method. The constructed libraries were sent to Vazyme Biotech company (Nanjing, China) and sequenced on Illumina HiSeq™ 2500 platform [35].

### Reads mapping and differential expression analysis

Raw reads obtained from the Hiseq 2500 platform were processed to filter out adapters, shorter reads and low quality reads. The resulting reads (clean reads) were mapped onto the tomato reference genome [36] using HISAT [37]. The gene expression levels were quantified by Expectation Maximization (RSEM) and Fragment Per Kilobase per Million mapped (FPKM) methods [38,39].

Differentially expressed genes (DEGs) were determined using NOISeq method (R/Bioc package) with a Noisy Distribution Model and shown by a Volcano diagram [40]. The screening criteria of a significantly enriched gene is as follows: a divergence probability > 0.8 and log2 fold change > 2.

### Gene annotation and enrichment analysis

Blast2GO software was used to predict the Gene Ontology (GO) annotation for all the unigenes. Functional annotation of all the unigenes was carried out using the WEGO software. The Kyoto Encyclopedia of Genes and Genomes (KEGG) metabolic pathway and signaling annotation was performed using KOBAS software. For the enrichment analysis, the significantly enriched GO and KEGG terms were selected. The differences of the assignment frequency of the GO or KEGG terms in the DEG pool were compared with all the expressed genes with *P* value < 0.05.

### Protein-protein interaction (PPI) network analysis

All DEGs were translated to protein sequences for the PPI network analysis. The resulting proteins were searched against the STRING database version 10.0 (http://string-db.org/). All interactions with a confidence score < 0.7 were fetched and visualized by Cytoscape software. A graph of the oretical clustering algorithm and molecular complex detection (MCODE) was utilized to analyze densely connected regions.

### Real-time PCR validation

In total, 1.0 μg of RNAs were sued to generate cDNAs using a cDNA synthesis kit (Invitrogen, Shanghai, China). The primer sequences used in qRT-PCR were designed using the Primerprimer 5 software and listed in **S1 Table**. The *Slactin* gene was used to analyse the relative fold differences based on the comparative cycle threshold values (2^-ΔΔCt^). The qRT-PCR was performed as follows: 1 μL of a 1/10 dilution of cDNA in ddH2O was add to 5 μL of 2× SYBR Green buffer (Takara, Dalian, China), 0.1 μM of each primer and ddH2O was then added to a final volume of 10 μL. The PCR conditions were 95°C for 10 min, 40 cycles of 95°C for 15 s and 60°C for 60 s.

### Statistical analyses

Differences in values between different groups were calculated using one-way analysis of variance with Student's *t*-test at *P* < 0.05 using the Excel software with ‘Analysis ToolPak’ add-in program. All expression analyses were performed basing on three biological repeats and figures show the average values of three repeats.

## Results

### Transcriptome sequencing

A total of 564.8 million raw reads were produced. Among the raw reads, about 564.3 million (99.9%) qualified reads were obtained for further analyses. Among the clean reads, 529,854,949 reads (approximately 93.9%) were mapped and 446,508,794 reads (about 79.1%) were unique mapped onto the tomato reference genome. Over 97.5% of the clean reads have quality scores at the Q20 level and over 93.9% of the clean reads have quality scores at the Q30 level (**S2 Table**). According to the reference genome, the proportion of reads mapped onto exons was 88.6%, onto introns was 3.6% and onto intergenic regions was 7.9% (**S3 Table**).

### Screening and classification of the genes responsive to hormone treatments

**Fig 1:**
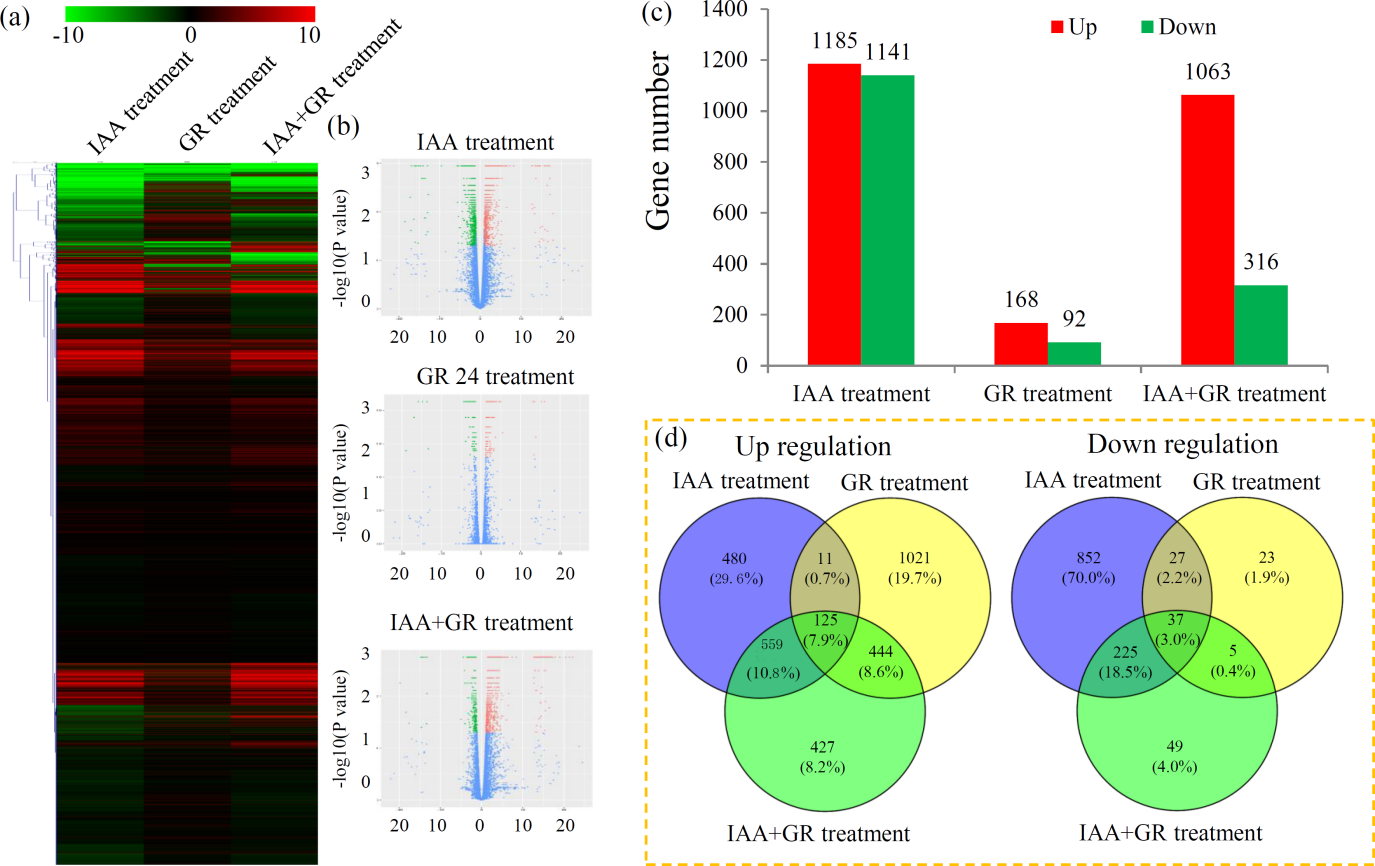
Transcriptional variations in tomato shoots under different hormone treatments. (a) Expression profiles of the DEGs under different hormone treatments were shown by a heatmap. (b) Significance analysis of the DEGs in different comparisons by Volcanoplots. (c) The number of up- and down-regulated genes in different comparisons. (d) Venn diagrams showed the proportions of the up- and down-regulated genes in three comparisons.

Transcriptional responses to the hormone treatments were determined by comparing the transcriptomes from the control and treated groups. Global gene expression profiles under different treatments are shown by a heatmap (**Fig 1A**). Under the IAA treatment, a total of 2326 DEGs, including 1185 up- and 1141 down-regulated genes, were identified. Under the GR24 treatment, 260 DEGs, including 168 up- and 92 down-regulated genes, were identified. Under the IAA+GR24 treatment, 1379 DEGs, including 1063 up- and 316 down-regulated genes, were identified (**Fig 1B** and **1C**). For the up-regulated genes, 480 genes were only induced under the IAA treatment, 1021 genes were only induced under the GR24 treatment, and 427 genes were only induced under the IAA+GR24 treatment. For the down-regulated genes, 852 genes were only reduced under the IAA treatment, 23 genes were only reduced under the GR24 treatment, and 49 genes were only reduced under the IAA+GR24 treatment (**Fig 1D**).

**Fig 2:**
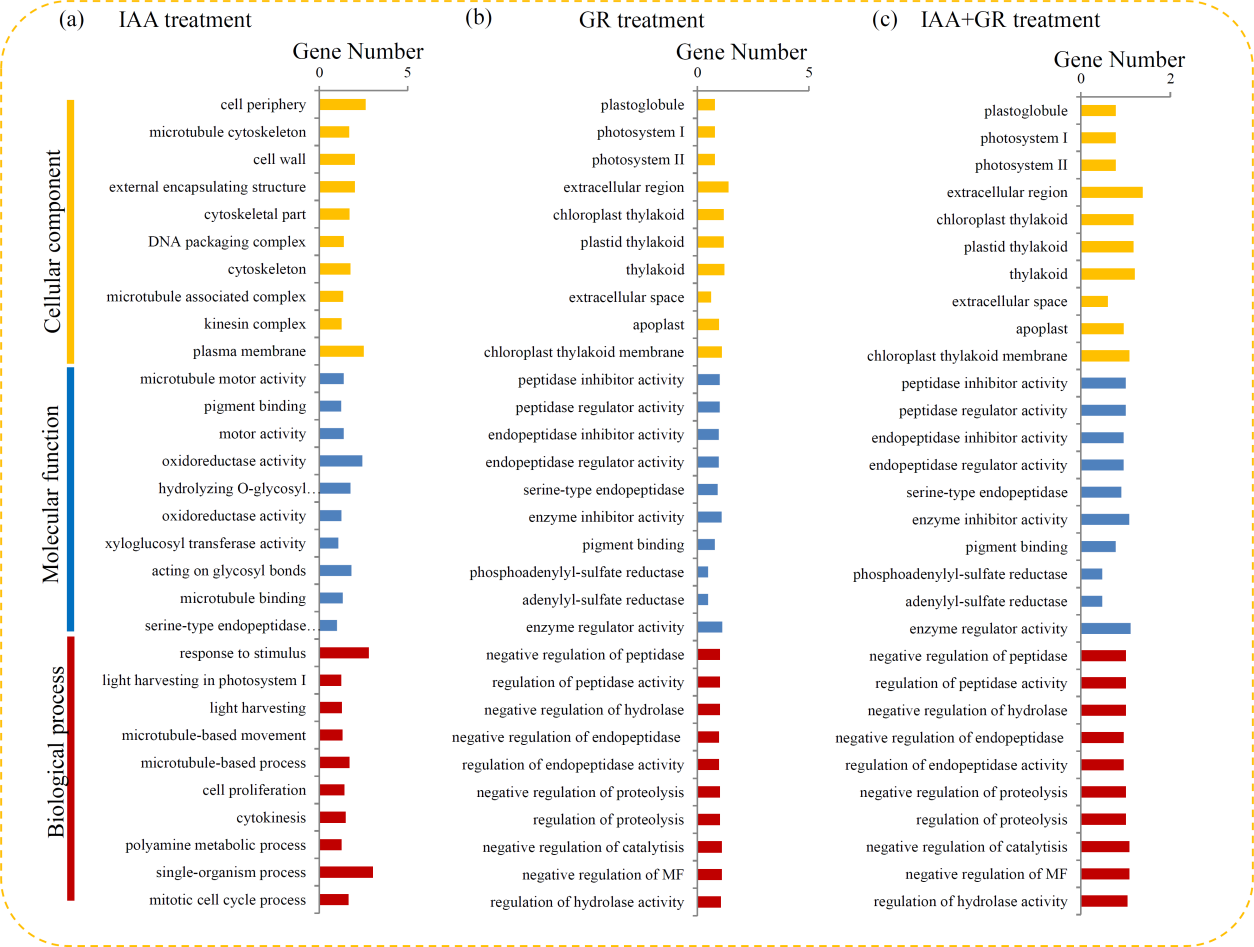
GO enrichment analysis of the DEGs in different comparisons. (a) Classification of the enriched GO terms under the IAA treatment. (b) Classification of the enriched GO terms under the GR24 treatment. (c) Classification of the enriched GO terms under the IAA+GR24 treatment.

To uncover their biological functions, the DEGs were annotated by GO-based sequence homologies. Under the IAA treatment, the top three largest GO terms in the biological process were “single-organism process”, “response to stimulus” and “microtubule-based process”; in the cellular component, the top three biggest GO terms were “cell periphery”, “plasma membrane” and “cell wall”; and in the molecular function, “oxidoreductase activity”, “acting on glycosyl bonds” and “hydrolyzing O-glycosyl compounds” were the top three largest GO terms (**Fig 2A**). Under the GR24 treatment, the largest GO terms in the biological process were “negative regulation of catalytisis” and “negative regulation of MF”; in the cellular component, the biggest GO terms were “extracellular region” and “thylakoid”; and in the molecular function, “enzyme regulator activity” and “enzyme inhibitor activity” were the largest GO terms (**Fig 2B**). Under the IAA+GR24 treatment, the top three largest GO terms in the biological process were “response to stimulus”, “response to chemical” and “response to organic substance”; in the cellular component, the top three biggest GO terms were “cell periphery”, “plasma membrane” and “extracellular region”; and in the molecular function, “oxidoreductase activity”, “sequence-specific DNA binding” and “nucleic acid binding” were the top three largest GO terms (**Fig 2C**).

### Network analysis of the DEGs under various treatments

**Fig 3:**
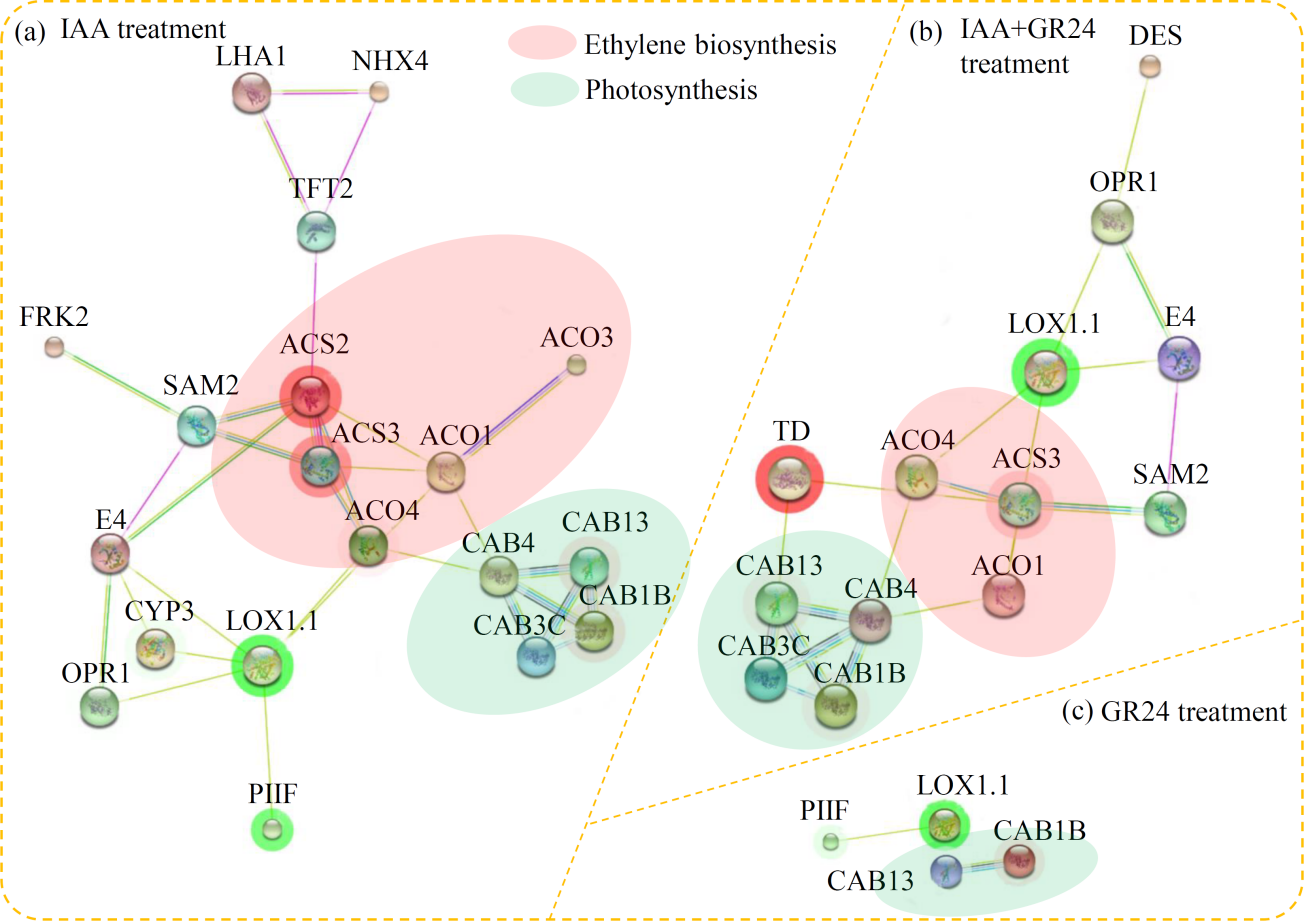
Interaction networks of the DEGs analyzed by Cytoscape software ver. 3.0.1. (a) The PPI network under the IAA treatment. (b) The PPI network under the IAA+GR24 treatment. (c) The PPI network under the GR24 treatment. Red background colour indicated the proteins involved in the ethylene biosynthesis pathway. Green background colour indicated the proteins involved in the photosynthesis pathway.

PPI network analysis was used to investigate the relationship among the DEGs. PPI networks of the DEGs under various hormonal treatments were examined. In total, 19, 13 and four DEGs were identified as network nodes under the IAA, GR24 and IAA+GR24 treatments, respectively. Furthermore, two enriched interaction clusters, including “ethylene biosynthesis” and “photosynthesis”, were identified in the PPI networks. In total, five and three ethylene biosynthesis-related genes were identified under the IAA and IAA+GR24 treatments, respectively, and four photosynthesis-related genes were identified under both IAA and IAA+GR24 treatments (**Fig 3**).

### KEGG enrichment analysis of the DEGs

**Fig 4:**
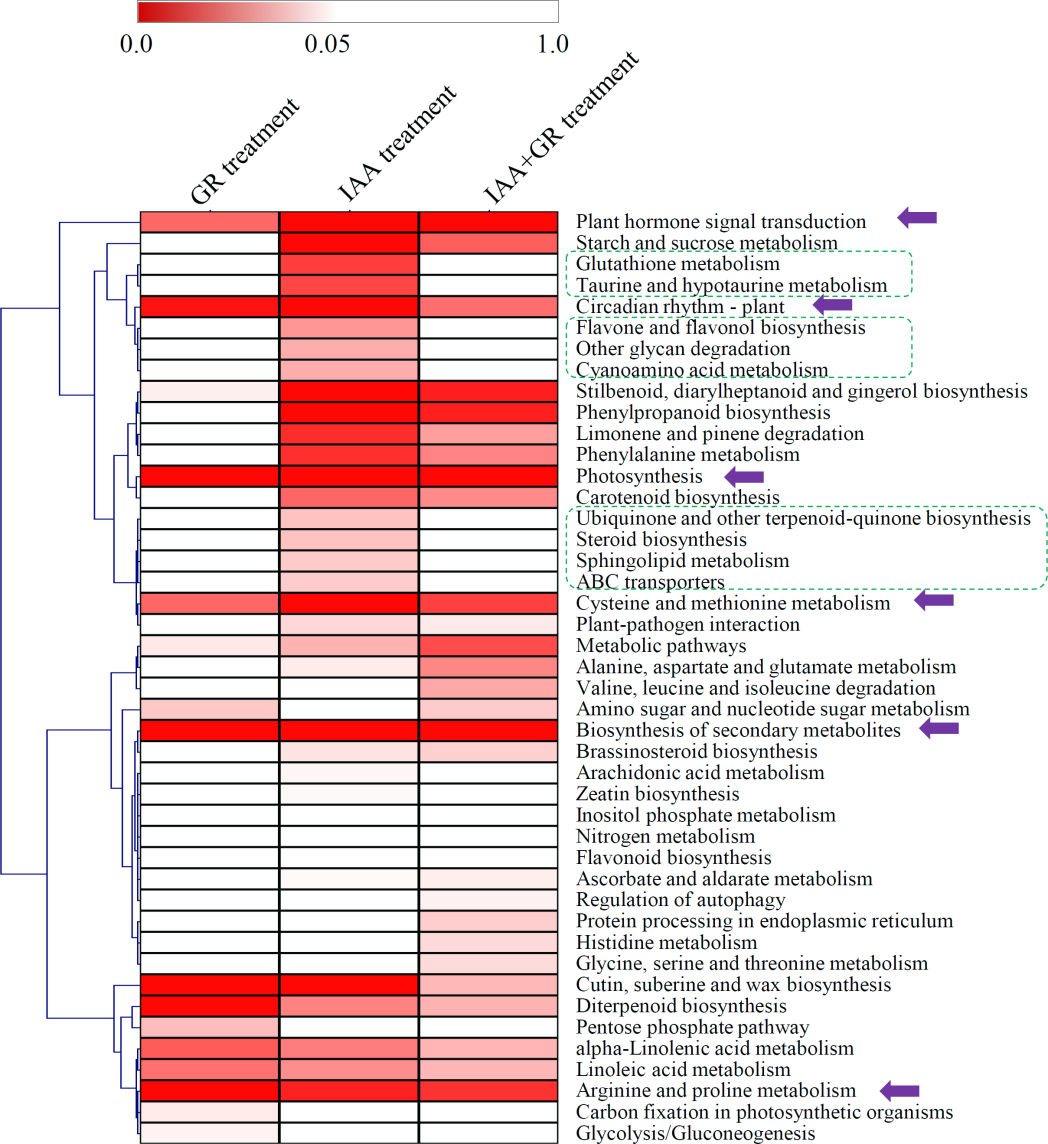
KEGG enrichment analysis of the DEGs in the three comparisons. The significant *P* values of each KEGG term under different hormone treatments were shown by a heatmap. Purple arrows indicated the metabolic pathways significantly changed under all the three treatments. Green boxes indicated the metabolic pathways only significantly changed under the IAA treatment.

A number of DEGs were classified into various KEGG signaling and metabolic pathways (**Fig 4**). Under the IAA treatment, the DEGs were significantly enriched in 27 KEGG pathways; under the GR24 treatment, the DEGs were significantly enriched in 12 KEGG pathways; and under the IAA+GR24 treatment, the DEGs were significantly enriched in 23 KEGG pathways (*P* < 0.05). Interestingly, six KEGG pathways, including “plant hormone signal transduction”, “circadian rhythm-plant”, “photosynthesis”, “cysteine and methionine metabolism”, “biosynthesis of secondary metabolites”, and “arginine and proline metabolism”, were enriched under the three treatments. Besides, several pathways, such as “glutathione metabolism”, “taurine and hypotaurine metabolism”, “flavone and flavonol biosynthesis”, “other glycan degradation”, “cyanoamino acid metabolism”, “ubiquinone and other terpenoid-quinone biosynthesis”, “steroid biosynthesis”, “sphingolipid metabolism”, and “ABC transporters”, showed significantly changes under the IAA treatment rather than under the GR24 and IAA+GR24 treatments (**S4 Table**).

### Analysis of the DEGs related to auxin signaling pathway

**Fig 5:**
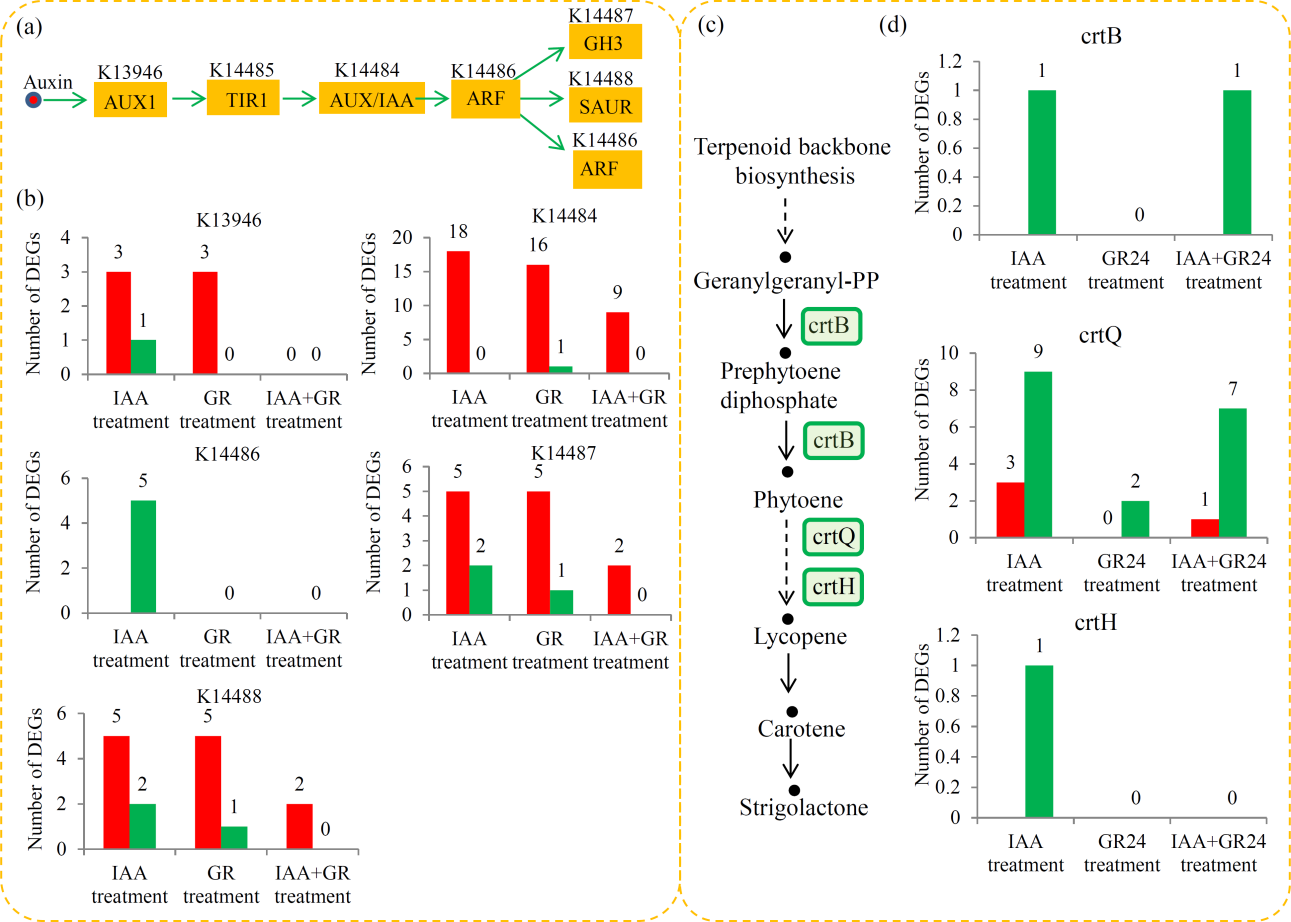
Transcript abundance changes of auxin- and SL-related genes. (a) Overview of the auxin signaling pathway in tomato. (b) The numbers of the DEGs involved in the auxin signaling pathway. (c) Overview of the SL biosynthesis pathway in tomato. (d) The numbers of the DEGs involved in the SL biosynthesis pathway.

Auxin levels were reported to be closely associated with the shoot-based grafting [7]. To explore the responses of tomato shoots to auxin, the expression of the genes involved in the auxin signaling pathway was analyzed. An overview of the auxin signaling pathway in tomato is shown in **Fig 5A**. For the auxin signaling pathway, a number of *AUX1* (K13946), *TIR1* (K14485), *AUX/IAA* (K14484), *GH3* (K14487) and *SAUR* (K14488) genes were identified (**Fig 5B**). The number of the auxin-related DEGs under the IAA and GR24 treatments was larger than that under the IAA+GR24 treatment. For example, three *AUX1* genes were up-regulated under both the IAA and GR24 treatments, and no significantly changed *AUX1* genes were observed under the IAA+GR24 treatment. Moreover, five *ARF* genes were down-regulated under the IAA treatment, and no significantly changed *ARF* genes were observed under both the GR24 and IAA+GR24 treatments.

### Analysis of the DEGs related to the SL biosynthesis pathway

SLs are derived from carotenoids, which are biosynthesized by the carotenoid biosynthetic pathway [41]. In our study, several DEGs involved in the carotenoid biosynthetic pathway were identified (**Fig 5C**). In detail, one *crtB* gene was significantly down-regulated under the IAA and IAA+GR24 treatments. For the *crtQ* genes, three up- and nine down-regulated genes were identified under the IAA treatment; two down-regulated genes were identified under the GR24 treatment; and one up- and seven down-regulated genes were identified under the IAA+GR24 treatment. Only one *crtH* gene was significantly reduced under the IAA treatment (**Fig 5D**).

### Validation of the expression of several key hormone-related genes

**Fig 6:**
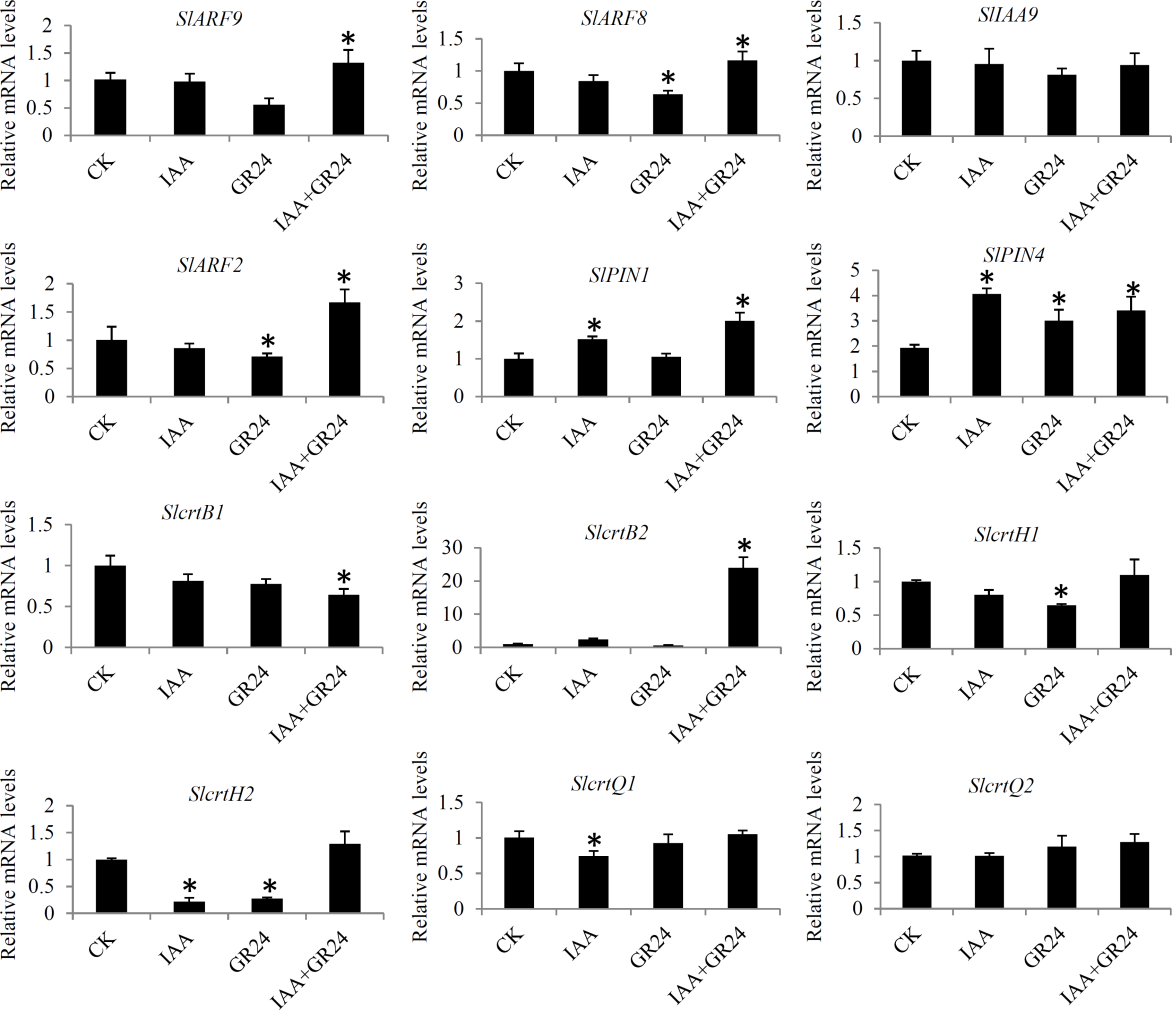
Real-time quantitative PCR validation of several hormone-related genes. The histogram shows the relative expression level of these genes with respect to the ACTIN in tomato. The data were analyzed by three independent repeats, and standard deviations were shown with error bars. Significant differences in expression level were indicated by “*”.

To verify the expression levels of some DEGs identified by RNA-seq, a qRT-PCR assay with independent RNAs collected from the same samples was applied. In total, 16 key hormone-related genes were randomly selected to check the data of the RNA-seq. Expression profiles of the selected genes under various hormonal treatments are showed in **Fig 6**. The expression levels of the selected genes were basically consistent with RNA-Seq results.

## Discussion

Phytohormones play essential roles in various biological processes in plants, such as plant immunity, abiotic stress tolerance, defense response and growth regulation [42–44]. Among the phytohormones, auxin and its signaling pathway have been well-studied in tomato [45]. For example, transcriptomic analyses suggested an alteration of the auxin pathway in tomato under the *Ralstonia solanacearum* infection [46]. A number of auxin response factors were reported to be involved in the fruit ripening process of tomato [47]. Besides, SLs also play important roles in various biological processes. Recent genetic evidences suggested a general role of SLs as messengers to suppress the lateral shoot branching in tomato [21]. SLs are positive regulators of several light-harvesting genes in tomato [48]. However, the interactions between auxin and SLs are largely unknown in tomato. DEG analysis is a powerful tool for studying on the temporal changes in gene expressions. In the present study, transcriptome data from the tomato shoots under various hormonal treatments was used to investigate the crosstalk between auxin and SLs.

Increasing evidences have revealed the interaction between auxin and SLs in plants. In the model plant *Arabidopsis*, SLs act downstream of the auxin signaling pathway to regulate novel shoot-branching and bud outgrowth [31]. SLs, the negative regulators of auxin polar transport, regulate the rice shoot gravitropism by decreasing auxin biosynthesis [49]. In pea mycorrhizal symbiosis, the auxin contents in roots were correlated with SLs exudation [50]. SLs mediate the regulation of somatic embryo formation and morphogenesis of tomato seedling through the crosstalk with auxin [27]. In our study, the numbers of DEGs greatly varied among the three treatments. Only 125 and 37 genes were commonly up- and down-regulated, respectively, under the three treatments. Our data confirmed a deep crosstalk between auxin and SLs in tomato shoots.

As a model plant for fruit ripening studies, the ethylene signaling pathway has been completely revealed in tomato. A number of ethylene synthesis and metabolism-related genes, such as *1-Aminocyclopropane-1-Carboxylate Oxidase* (ACO), *Ethylene Insensitive 2* (EIN2), *1-Aminocyclopropane-1-Carboxylate* (ACC) and *ACC synthase* (ACS), have been functional identified in tomato [51–53]. In our study, PPI analysis also pointed out an enrichment cluster related to ethylene biosynthesis under the IAA and IAA+GR24 treatments (**Fig 3**), suggesting that auxin was involved in the regulation of ethylene biosynthesis [54]. In tomato, auxin signaling and auxin accumulation are required for the systemic enhancement of photosynthetic induction [55]. GR24 treatment significantly increased the photosystem II quantum yield in rice [56]. Interestingly, four photosynthesis-related genes, including *Chl A/B-Binding protein* (CAB) *1B*, *CAB13*, *CAB3C* and *CAB4*, were up-regulated by the IAA and IAA+GR24 treatments and two photosynthesis-related genes, including *CAB13* and *CAB1B*, were up-regulated by the GR24 treatment. Our data suggested that auxin-SLs-triggered expressions of several *CAB* genes may lead to the systemic increases in the photosynthesis induction.

Both auxin and SLs have been reported to be involved in the regulation of metabolic pathways. Auxin affects the homeostasis of a series of metabolic pathways during the grafting process of *T*. *grandis* [57]. A iTRAQ-based quantitative proteomics revealed that the GR24-regulated proteins in *Arabidopsis* were involved in various metabolic processes [58]. Our data showed that several auxin-activated metabolic pathways, such as the flavone and flavonol biosynthesis and steroid biosynthesis pathways, were inhibited by the auxin+GR24 combined treatment. It suggested that the crosstalk between auxin and SLs might be involved in the regulation of metabolic pathways in tomato.

SLs manipulate the auxin pathway by affecting the cellular trafficking, perception and downstream responses of auxin [33]. For example, SL signalling enhances the turnover of PIN1 on plasma membrance and increases the plasma-membrane localization of PIN2 [59]. In our study, a large number of auxin downstream responsive genes, including 16 *AUX/IAA* genes, five *GH3* genes, and five *SAUR* genes, were up-regulated by the GR24 treatment (**Fig 5**). SLs affect the auxin responses of tomato shoots by up-regulating the expression of a series of auxin downstream genes, suggesting that the biosynthesis of SLs was conducted by auxin. In tomato, the biosynthesis of SLs was controlled by IAA27, a key regulator of the auxin responses [34]. Interestingly, the expression of several genes related to the “Carotenoid biosynthesis” pathway, which provided precursors for the SLs biosynthesis, was significantly changed by the IAA treatment, indicating that auxin regulated the biosynthesis of SLs by affecting the “carotenoid biosynthesis” pathway.

In conclusion, we explored the transcriptomic changes in tomato shoots under various hormonal treatments. Four independent groups of cDNA libraries from the control, IAA-treated, GR24-treated and IAA+GR24-treated shoots of tomato seedlings were separately sequenced. A great number of DEGs were identified in different comparisons, including the IAA vs control, GR24 vs control, and IAA+GR24 vs control comparisons. The crosstalk between auxin and SLs may be involved in the regulation of metabolic pathways in tomato. SLs affect the auxin responses of tomato shoots by up-regulating the expression of a series of auxin downstream genes. Auxin regulated the biosynthesis of SLs by affecting the “carotenoid biosynthesis” pathway. Our data will give us an opportunity to reveal the crosstalk between auxin and SLs in tomato shoots.

## Supporting information

S1 Table The primer sequences of the PCR verification. (XLSX)

S2 Table Summary of sequencing results of tomato samples under different treatments. (XLSX)

S3 Table Alignment distribution of the unique mapped reads. (XLSX)

S4 Table The significant values of each KEGG pathway under different treatments. (XLSX)
