# Correction: Transcriptome analysis of tomato (*Solanum lycopersicum* L.) shoots reveals a crosstalk between auxin and strigolactone

**DOI:** 10.1371/journal.pone.0204873

**Published:** 2018-09-25

**Authors:** Yihua Zhan, Yinchao Qu, Longjing Zhu, Chenjia Shen, Xuping Feng, Chenliang Yu

The Data Availability statement for this paper is incorrect. The correct statement is: The data underlying this study have been uploaded to the Sequence Read Archive and are available using the following accession number: SRP131701.
